# Mediators and moderators of nutrition intervention effects in remote Indigenous Australia

**DOI:** 10.1017/S0007114518000880

**Published:** 2018-06-28

**Authors:** Julie Brimblecombe, Megan Ferguson, Federica Barzi, Clare Brown, Kylie Ball

**Affiliations:** 1 Department of Nutrition, Dietetics and Food, School of Clinical Sciences, Faculty of Medicine, Nursing and Health Sciences, Monash University, level 1, 264 Ferntree Gully Road, Notting Hill, VIC 3168, Australia; 2 Menzies School of Health Research, Wellbeing and Preventable Chronic Diseases Division, Royal Darwin Hospital Campus, Rocklands Drive, Tiwi, NT 0810, Australia; 3 School of Exercise and Nutrition Sciences, Institute for Physical Activity and Nutrition (IPAN), Locked Bag 20000, Geelong, VIC 3220, Australia

**Keywords:** Diets, Mediators, Moderators, Indigenous communities, Dietary intakes, Longitudinal sub-studies

## Abstract

We conducted a longitudinal dietary intervention study to assess the impact of a store-based intervention on mediators and moderators and consequent dietary behaviour in Indigenous communities in remote Australia. We assessed dietary intake of fruit, vegetable, water and sweetened soft drink, mediators and moderators among 148, eighty-five and seventy-three adult participants (92 % women) at baseline (T1), end of intervention (T2) and at 24 weeks post intervention (T3), respectively. Mediators included perceived affordability and self-efficacy. Moderators were barriers to eat more fruit and vegetables and food security. Mixed-effects models were used to determine changes in mediators and moderators with time and associations between these and each dietary outcome. Perceived vegetable affordability increased from T1 (19 %; 95 % CI 11, 27) to T2 (38 %; 95 % CI 25, 51) (*P*=0·004) and returned to baseline levels at T3. High self-efficacy to eat more fruit and vegetables and to drink less soft drink decreased from T1 to T3. A reduction in soft drink intake of 27 % (95 % CI −44, −4; *P*=0·02) was reported at T3 compared with T1; no changes with time were observed for all other outcome measures. Regardless of time, vegetable intake was positively associated with self-efficacy to cook and try new vegetables, no barriers and food security. The dietary intervention went someway to improving perceived affordability of vegetables but was probably not strong enough to overcome other mediators and moderators constraining behaviour change. Meaningful dietary improvement in this context will be difficult to achieve without addressing underlying constraints to behaviour change.

Diet is the leading contributor to the global burden of disease^(^ ^1^ ^)^. Despite improvements in diet in some populations in recent years, a dietary social gradient exists in high-income countries^(^ ^2^ ^–^ ^5^ ^)^. Australians from socio-economically disadvantaged backgrounds tend to consume a poorer quality^(^ ^6^ ^)^ and less varied diet^(^ ^7^ ^)^, less fruit and vegetables^(^ ^6^ ^,^ ^8^ ^)^ and more energy-dense nutrient-poor ‘discretionary’ foods^(^ ^9^ ^)^ than more affluent Australians. Aboriginal and Torres Strait Islander Australians (respectfully hereafter referred to as Indigenous Australians), who before European colonisation managed and sustained their food environment to meet their needs, are now among the most socially disadvantaged Australians. Adverse diet is a major contributor to the excessive burden of ill health experienced by Indigenous Australians^(^ ^10^ ^–^ ^12^ ^)^.

In putting in place effective and comprehensive strategies to support dietary improvement, the complexity of dietary behaviour^(^ ^13^ ^)^ needs to be considered. This complexity is distinctive in remote Australian communities where an array of factors influences dietary behaviour. There are over 160 discrete communities in very remote Indigenous Australia with populations of more than 100 people^(^ ^14^ ^)^. These communities are largely dependent on an imported food supply provided by the community store^(^ ^14^ ^)^. The geographic remoteness of these communities in combination with the limited buying power of stores and limited wholesale options are factors that drive the price of food up to more than 60 % higher than that in metropolitan centres^(^ ^15^ ^)^. Coupled with low income and other economic stressors, dietary quality and quantity for many people fluctuates from bad to worse across pay cycles^(^ ^16^ ^–^ ^18^ ^)^. Many households are over-crowded and/or have only rudimentary cooking and storage facilities, making home food preparation challenging^(^ ^19^ ^,^ ^20^ ^)^. In all, 31 % of Indigenous adults in remote Australia report food insecurity compared with 3·7 % for all Australians^(^ ^21^ ^)^.

However, community dietary patterns in this complex environment have been found to respond to multi-component interventions that have engaged community leaders and addressed determinants of behaviour at both the environment (e.g. through improving food availability and quality mostly via the community store) and individual levels (e.g. through behaviour change communication activities)^(^ ^22^ ^–^ ^24^ ^)^. It is important to measure the main effects of such interventions; however, there has been scant exploration of the mediators (how programmes effect change) and moderators (for whom programmes work best) that play a role in influencing dietary behaviour change in this context. Identifying these is important to understand the mechanisms and constraints to behaviour change so as to design strategies that can best effect change^(^ ^25^ ^)^.

The SHOP@RIC study was a multi-component, price discount and in-store consumer education-based intervention strategy that aimed to improve fruit, vegetable and water consumption and reduce sugar-sweetened soft drink (regular soft drink) consumption in remote Indigenous communities. It was informed by social–ecological and social cognitive theory^(^ ^26^ ^)^ and was conducted over a 24-week period in twenty very remote Aboriginal communities (ten receiving discount only and ten receiving both discount and consumer education)^(^ ^27^ ^)^. The price discount comprised a 20 % discount on fruit, vegetables, bottled water and artificially sweetened soft drinks (diet soft drinks) in community stores, and aimed to improve affordability and intake of these targeted foods and beverages. The consumer education component included a focus on enhancing self-efficacy, as limited nutrition literacy of the rapidly expanding imported food supply and skills to prepare these ‘new-comer’ foods are key factors influencing diet^(^ ^10^ ^,^ ^28^ ^)^.

Hence, perceived affordability and self-efficacy were hypothesised mediators (measures of how the programme affects change) of intervention effects in this study.

Similarly, given the substantial challenges to dietary improvement posed by the array of barriers present in Indigenous communities including food insecurity, it was hypothesised that these might moderate intervention effectiveness. We therefore assessed the barriers to individuals of high price, providing for a large number of people in the household, inadequate food storage and equipment for preparation, and food insecurity.

The sub-study reported here was thus planned to assess the impact of a store-based intervention on hypothesised mediators and moderators and consequent dietary behaviour among primary household shoppers living in the communities that received the combined price discount and consumer education strategy. The aims were to determine the following: (i) change in mediators over the course of the intervention and follow-up period; (ii) whether changes in mediators were associated with dietary change; and (iii) whether mediators/ moderators were associated with dietary intake regardless of time. We hypothesised that perceived affordability and/or self-efficacy would be higher at the end of the intervention compared with the baseline and that higher perceived affordability and/or self-efficacy at baseline would be associated with improvements in dietary intake at the end of the intervention and that these improvements in intake would be sustained post intervention.

## Method

### Study design

The sub-study reported here used a longitudinal dietary intervention design with the collection of baseline (T1), immediately before cessation of the 24-week intervention (T2) and 24-week post-intervention data (T3).

The larger SHOP@RIC study was a stepped wedge randomised trial; detail of its design is described elsewhere^(^ ^27^ ^)^. In brief, twenty consenting communities were randomly grouped into five sets of four communities, and each set was randomly allocated to initiate the price discount at one of five possible time points (the first set commenced in June 2013), spaced 8 weeks apart. Two stores in each set were randomly allocated to receive the combined intervention (price discount and consumer education). This longitudinal sub-study was conducted among a cohort of community members recruited from five randomly selected communities, comprising one community receiving the combined intervention in each of the five store sets.

### Ethics

The SHOP@RIC study was approved by the Human Research and Ethics Committee (HREC) of the Northern Territory Department of Health and Menzies School of Health Research (HREC-2012-1711) and the Central Australian HREC (HREC-12-13). Each participating community store board provided written consent following presentation and discussion of the project to the store board. Community leaders were consulted in the five study communities and all agreed to participate in the longitudinal sub-study. Advice was sought in each community from the store board on the recruitment of at least two local community members who then received training on the study protocol and assisted the research team with recruitment, translation or interpretation of the survey questions, data collection and study feedback. Training of these personnel occurred over 3–5 d to prepare them for the role, and covered an overview of the study and basic nutrition concepts, the research process, ethics, consent, interview conduct and use of the iPad to record data. This training contributed to attaining a Certificate 11 in Health Research for those interested. The community researchers involvement helped ensure that study processes and the conduct of research team members were appropriate to the cultural practices of the community^(^ ^29^ ^)^. Individual participants provided written consent.

### The intervention

The intervention was a combined price discount and consumer education strategy, informed by social–ecological and social cognitive theories^(^ ^27^ ^)^. The price discount was promoted at front-of-store and at product location. The consumer education strategy aimed to be consistent with national and local nutrition guidelines, engaging and culturally appropriate for Indigenous people living in remote communities, and sustainable in terms of being low cost, practical and standardised across the ten participating communities. It was developed using intervention mapping^(^ ^30^ ^)^, which involved a six-step process that first involved mapping barriers to relevant mediators, and resulted in a matrix of behaviour change objectives, performance objectives and behaviour change techniques. This then informed the development of six themes (one theme implemented every 4 weeks) and a set of accompanying activities that included a poster and activity sheet for each theme, two fridge stickers, one cooking demonstration, one taste-testing, a sugar-in-drinks display and a receipt competition.

### Setting and participants

The five communities from whom the data for this sub-study were collected ranged in size from approximately 200 to 400 residents and were located across the Northern Territory of Australia. The aim was to recruit approximately 200 Aboriginal adults who identified as a primary shopper for the household: at least forty from each of the five communities, allowing for an estimated attrition of 25 % to have a total sample of 150 at T3. This sample size was determined based on the number of communities for which it was economically feasible to visit three times within the study period and the number of participants deemed to be achievable in these small communities where household overcrowding is persistent. Estimated attrition was based on the successful retention rates experienced by Sayers *et al.* ^(^ ^31^ ^)^ in the Aboriginal Birth Cohort study. Using a community map, all households in the community were numbered and a list of households to invite to participate was made by randomly selecting household numbers from a hat until forty households had been selected. Where there were less than forty households, a second ‘primary’ shopper (given that more than one family commonly resides in a house) was invited to participate according to the randomisation process. On a visit to each household, an adult meeting the eligibility criteria (i.e. community resident, plans to reside in the community for 12 months, ≥18 years, purchases food from the community store and is the primary shopper) was nominated by the household or self-nominated generally in the presence of other household members, and then invited to participate in the study following explanation of its purpose. Where interest was expressed, a suitable interview time and place was arranged. The primary shopper was invited to participate in a pre-intervention (within the 4 weeks preceding the intervention/T1), end of intervention (in the last 2 weeks of the 24-week intervention/T2) and post-intervention interview (24 weeks after the intervention/T3). In the case of a decline to participate at T1, unless a second primary household shopper was nominated, the team moved on to the next household. On completion of the interview at each of the three time points, a $20 gift of fruit, vegetables and water was provided to each participant.

### Measures

A 51- to 63-item questionnaire (depending on time point) was developed to collect self-reported information at the three time points (T1, T2 and T3). The questionnaire was completed by the participant via an iPad with the assistance of a member of the research team and according to standardised procedures, including scripted introductions and explanations where required.

Demographic information was collected on age group, sex, employment, education and number of people that the participant shopped for. Outcome measures included daily intake of fruit (g), vegetable (g), water (ml), regular soft drink (ml) and diet soft drink (ml). Frequency consumption (i.e. never, once/fortnight, 1 d/week, 2–3 d/week, on most days and everyday) of fruit and vegetables (including fresh, frozen, dried and canned) and water and diet and regular soft drink and amount (g) usually consumed were assessed. Daily self-reported intakes were derived. As fruit and vegetable intake in remote communities in Australia is known to be low^(^ ^32^ ^)^ and we expected less than a serve (i.e. 30 g) increase per person in fruit and vegetables combined per day as a result of the SHOP@RIC intervention^(^ ^27^ ^)^, we used a methodology that would quantify daily intake in grams. The facilitated self-report methodology developed was tested with sixteen Aboriginal residents across three remote Indigenous communities.

There is a lack of validated dietary assessment methods for this population, and there are specific challenges to assessing diet in Aboriginal populations, including typically low levels of school-based education, literacy and numeracy, and cultural considerations^(^ ^33^ ^)^ (e.g. direct questioning techniques are not typically practiced in traditional Indigenous culture). On the basis of pilot testing, a script and pictorial aides were developed to assist respondents. A similar facilitated self-report methodology was used to estimate intakes of water, diet drinks and regular soft drink.

Daily intakes (g/ml) were then derived by multiplying frequency (in d) by usual amount consumed, where once per fortnight was imputed as 0·5 d/week, 1 d/week as 1 d, 2–3 d as 2·5 d, most days as 5 d and everyday as 7 d/week. A fortnightly calendar showing days of significant events occurring in the community, such as pay day or food delivery to the store, was used to assist respondents determine frequency consumption. An estimate of the amount usually consumed was aided by use of visuals showing common serving sizes.

The full set of mediator and moderator variables are shown in the online Supplementary Table S1 with questions and re-coding. These were assessed at all three time points. An instrument to assess mediators used among non-Indigenous adult consumers in the SHELf study^(^ ^25^ ^)^ who were exposed to a similar intervention to that of the SHOP@RIC study was identified and modified through pilot testing to ensure face validity and relevance to the study population^(^ ^25^ ^)^. The mediators assessed included perceived affordability of fruit/ vegetables; self-efficacy to positively change intake; self-efficacy to cook and try new vegetables; and perceived new knowledge (fruit/vegetables/drinks). Data on perceived new knowledge were collected at T2 only and therefore were not included in the mediator analysis. Other mediators (i.e. outcome expectancies) associated with social cognitive theory were included at T1 but not continued owing to near-universal positive responses. The survey variables were combined into summary variables to use in the analysis as shown in the online Supplementary Table S1 to minimise multiple comparisons and owing to co-linearity of similar variables. Mediator variables with nominal response options were recoded to have only two categories (yes and no) owing to small numbers. Data were also collected on hypothesised moderators – barriers to increasing fruit and vegetable intake (as described earlier and informed by a study conducted in one remote Aboriginal community in the NT^(^ ^28^ ^)^) and household food security (using the standardised measure applied in the National Aboriginal and Torres Strait Islander Health Survey^(^ ^21^ ^)^). An additional question that related to food security based on previous formative research^(^ ^28^ ^)^ was included (refer to the online Supplementary Table S1).

### Statistical analyses

Participants’ demographics, food and drink intakes, mediators and moderators were described for the overall sample at T1 and for the sub-samples of participants who completed the questionnaire at each of T2 and T3. No imputation of missing values was done owing to the large amount of missing data. Categorical variables were described as percentages, and continuous variables for food and water intake were log-transformed and summarised with mean and 95 % CI transformed back to the normal scale. Data on diet soft drink were not used in the analyses owing to low reported median intake (0 ml/p per d (interquartile range (IQR) 0–54) and 55 % of the population reporting no consumption at T1. As the distribution of regular soft drink intake could not be normalised, median and interquartile range were used to summarise the variable.

Percent changes in mediators and moderators from T1 to T2 and from T1 to T3, or when appropriate from T2 to T3, were estimated using mixed-effect logistic models. Associations between the log intake of each of fruit, vegetables and water and the dichotomous variables for mediators and moderators were estimated using mixed-effects linear models. Effects were expressed in terms of percent difference relative to the reference group. Similar models were used to look at the changes in intake over time (from T1 to T2 and from T1 to T3), overall and separately, according to each mediator and moderator level by including time by mediator (or moderator) interaction terms. A mixed-effect negative binomial model was used for regular soft drink intake and effects expressed in terms of percent difference relative to the reference group.

All the mixed models included a random intercept to account for within-subject correlation and community clustering effect to account for within-community correlation. The potential confounding effect of participants’ demographics was explored including variables for age, education and employment in each model. All the models used all the available data at each of T1, T2 and T3. Statistical analyses were performed using Stata version 14 (Stata Corporation)^(^ ^34^ ^)^.

## Results

A total of 148 participants (91 % aged 26 years and older, 92 % female, 80 % with at least some secondary education, 39 % employed) completed the baseline (T1) questionnaire; eighty-five and seventy-three participants also completed the T2 and T3 questionnaires, respectively. We did not reach our target of 200 at baseline owing to the small size of the communities randomly selected. Fewer young people (≤26 years) participated in T2 and T3 surveys compared with the baseline. No other differences in demographics, intake, mediators or moderators between completers and non-completers were observed. Twenty participants declined invitation to participate in T2 or T3. Other dropouts were because of the participant not being present in the community at the time of the survey, being unavailable or unable to be located. This is not unusual because of the remote Indigenous population being highly mobile. Participants shopped for around seven people on average (Table 1).

**Table 1:** Demographics for baseline (T1) and cohort at T2 and T3 (Numbers and percentages; mean values and standard deviations)

	T1 (*n* 148)		T2 (*n* 85)		T3 (*n* 73)	
	*n*	%	*n*	%	*n*	%
Age (years)						
18–25	14	9·5	3	3·5	5	6·9
26–35	39	26·4	20	23·5	18	24·7
36–45	39	26·4	24	28·2	21	28·8
46–55	30	20·3	18	21·2	16	21·9
56+	26	17·6	20	23·5	13	17·8
Sex						
Female	136	91·9	80	94·1	71	97·3
Employment						
Full-time employed	28	18·9	16	18·8	14	19·2
Part-time employed	29	19·6	19	22·4	18	24·7
Not employed	63	42·6	30	35·3	32	43·8
Retired/other	28	18·9	20	23·5	9	12·3
Education						
Primary at most	29	19·6	19	22·4	14	19·2
Some secondary	85	57·4	51	60·0	46	63·0
Year 12 and above	34	23·0	15	17·7	13	17·8
Number of people shopping for						
Mean	6·8		6·8		7·3	
sd	3·0		2·6		4·2	

### Dietary intake (baseline)

**Fig. 1:**
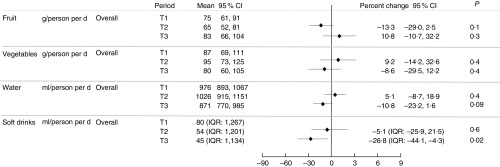
Self-reported intake of fruit, vegetable, regular soft drink and water per person per day by period (T1, T2 and T3) and percent change from T1 to T2/T3. IQR, interquartile range.

At T1 the mean daily per person intake was 75 g (95 % CI 61, 91) for fruit and 87 g (95 % CI 69, 111) for vegetables (Fig. 1). Five respondents met dietary recommendations for fruit (≥300 g/d) and thirteen for vegetable intake (≥375 g/d) (data not presented). Water intake was estimated at 976 ml (95 % CI 893, 1067) per person per day on average. The median intake for regular soft drink was 80 ml (IQR 1, 267) per person per day (Fig. 1) and 152 ml (IQR 54–375) per person per day including only participants who reported an intake >0 ml/d at least at one time point (data not presented). A reduction in soft drink of 27 % (IQR −44·1, −4·3; *P*=0·02) was shown at T3 compared with T1. Small changes were observed with time for the other outcome measures; however, we did not have adequate power to detect whether these were statistically significant (Fig. 1).

### Mediators/moderators (at baseline and over time)

**Fig. 2:**
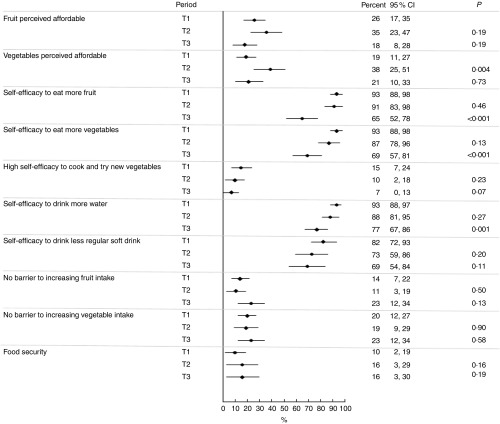
Percentage of participants reporting mediators by period (T1, T2 and T3).

At T1, as shown in Fig. 2, less than one-third of participants perceived fruit (26 %) and vegetables (19 %) as affordable, whereas the majority reported high self-efficacy to eat more fruit (93 %), more vegetables (93 %), to drink more water (93 %) and to drink less regular soft drink (82 %). Less than one-quarter (15 %) reported high self-efficacy to cook and try new vegetables, and 14 and 20 % reported no barriers to eating more fruit and more vegetables, respectively (i.e. they reported to ‘eat enough’). One in ten participants (10 %) reported to be food secure. The online Supplementary Table S2 shows responses at each of the time points for all mediator and moderator variables.

Perceived affordability of vegetables (*P* 0·004) improved at T2 and a non-significant increase was shown for fruit at T2. However, these improvements did not persist at T3 (Fig. 2). Self-efficacy to consume more fruit (*P*<0·001), vegetables (*P* <0·001) and water (*P*=0·001) and to cook and try new vegetables (*P*=0·07) was lower at T3 compared with T1, and did not differ between T1 and T2. A similar non-significant trend was observed for self-efficacy to drink less regular soft drink.

### Association between intake and mediators/moderators at any time

As shown in the online Supplementary Table S3, vegetable intake overall was 77 % higher (95 % CI 32 %, 123 %; *P*=0·001) in those with high self-efficacy to cook and try new vegetables relative to those with low self-efficacy to cook and try new vegetables and 53 % (95 % CI 17, 89; *P*=0·004) and 89 % (95 % CI 36, 143; *P*=0·001) higher among those with no barriers and food security, respectively, compared with those with barriers/food insecurity. No other mediators were associated with vegetable intake and no statistically significant associations with mediators or moderators were shown for fruit, water and regular soft drink intake.

### Associations between mediators/moderators and changes in intake

**Fig. 3:**
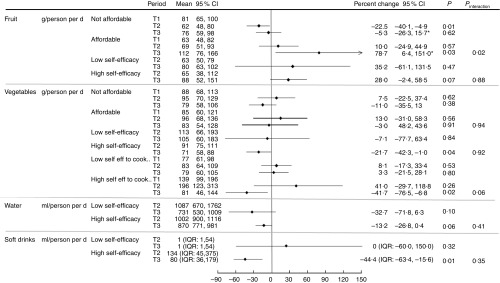
Percentage change in intake of fruit, vegetables, water and regular soft drink from T1 to T2/T3 and T2 to T3 (for self-efficacy), by mediator. *P<0·05 for interaction between mediator and period. IQR, interquartile range.

**Fig. 4:**
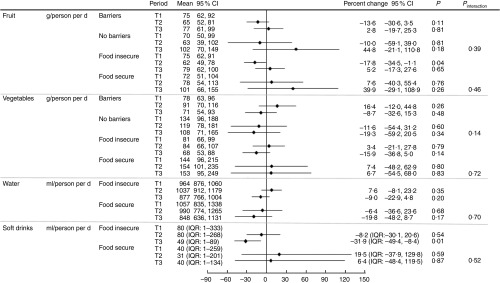
Percentage change in intake of fruit, vegetables, water and regular soft drink from T1 to T2/T3, by moderator. IQR, interquartile range.

Analysis of mediators and moderators showed that perceived affordability of fruit was associated with an improvement in dietary intake (Fig. 3 and 4). Those who perceived fruit to be affordable at T1 reported an increase in intake at T3, whereas those who perceived fruit not affordable at T1 showed no change at T3 (79 *v*. −5 %; *P* value difference for interaction= 0·02). There appeared to be some change in intakes at T2 and/or T3 compared with baseline in association with the mediators and moderators (Fig. 3 and 4) (e.g. those who reported high self-efficacy to cook and try new vegetables at T1 appeared to have higher reported intakes of vegetables at T2 compared with T1 than those with low self-efficacy); however, we did not have the power to detect whether these differences between the two groups were statistically significant.

Adjusting for covariates, such as age, education and employment, had little impact on the results for all analyses.

## Discussion

This novel study is the first we are aware of to provide evidence on how a combined price discount and consumer education strategy may effect change and for whom the programme may work best. This information can help in the design of future strategies to help improve diet. The strategy was successful in modifying perceived affordability of both fruit and vegetables while it was applied. This intervention effect was associated with an increase in fruit intake but only at 24 weeks post intervention. It did not seem to modify low self-efficacy to cook and try new vegetables, or further shift the already high self-efficacy to eat more fruit, vegetables and water reported at baseline. A reduction in regular soft drink intake was shown at 24 weeks post intervention despite there appearing to be no mediating or moderating effects for beverages. We did not have the power to detect whether there was an intervention effect on reported fruit or reported vegetable intake.

This study shows that those with high self-efficacy to cook and try new vegetables, no reported barriers and food security had a higher vegetable intake and that even towards the end of the intervention about two-thirds of the cohort still perceived fruit and vegetables, respectively, as not affordable, 90 % reported low self-efficacy to cook and try new vegetables, 84 % reported food insecurity (62 % using the standard measure alone) and 89 and 71 % reported barriers to consume more fruit and vegetables, respectively. These findings suggest that socio-economic constraints and limited self-efficacy to cook and try new vegetables prevented participants from being able to take advantage of the intervention and make a positive change in dietary intake and more importantly that the intervention was not strong enough or able to overcome these.

In contrast to our findings, no reduction in regular soft drink was detected for the larger SHOP@RIC trial^(^ ^35^ ^)^, suggesting that further investigation of effective approaches to reduce regular soft drink consumption is required. These findings also indicate that population-level data may not detect reduction if occurring in a sub-set of the population only.

Cessation of the intervention seemed to negatively affect perceived affordability of fruit and vegetables. This response is in contrast to what we hypothesised, but might be expected with the return to pre-intervention prices on removal of the price discount and indicates the price sensitivity of consumers in this context. The reduction observed in self-efficacy 24 weeks after cessation of the intervention is difficult to explain particularly, as no change was observed in self-efficacy at the end of the intervention. It may be that the intervention raised a level of awareness that resulted in people feeling less capable to modify their dietary behaviour than they thought they were.

In this study, the majority of participants were not in paid employment, and less than one-quarter had year 12 education or above. These statistics reflect the widely documented social disadvantage concentrated in remote Indigenous Australia. Contributing to this social disadvantage is the high cost of food^(^ ^15^ ^,^ ^36^ ^)^. Food affordability is one of the most common cited barriers to fruit and vegetable consumption among socio-economically disadvantaged populations^(^ ^28^ ^,^ ^37^ ^,^ ^38^ ^)^ and has been associated with consumption^(^ ^39^ ^)^. Perceptions of affordability are also probably influenced by other non-economic considerations rather than actual price alone (such as home storage and food preparation facilities) and/or that how people weight affordability is probably considered relative to the affordability of other foodstuffs and items. In a large cross-sectional study among women residing in neighbourhoods of low socio-economic advantage, perceptions of poorer food availability and quality (which are issues in remote Indigenous communities) were associated with lower perceived affordability. This suggests that consumers’ interpretation of their local food environment may also influence perceptions of food affordability and the types of food considered ‘value for money’^(^ ^39^ ^)^.

Self-efficacy to cook and try new vegetables was the only mediator associated with vegetable intake. Even though our study intervention did not enhance self-efficacy, those in the study cohort who had higher self-efficacy to cook and try new vegetables consumed more vegetables than those with lower self-efficacy. Therefore, enhancing cooking skills and confidence to try new foods may be an important focus area to improve dietary intake, particularly given that others have shown cooking skills and confidence to be positively associated with vegetable purchasing^(^ ^40^ ^)^ and healthier eating^(^ ^41^ ^,^ ^42^ ^)^. In one large community in very remote Australia, many residents possessed basic knowledge of the healthiness of store foods, and wanted to increase familiarity and experience with non-traditional foods through attaining practical skills, especially in cooking^(^ ^43^ ^)^. A number of programmes have reported success in enhancing cooking skills among Aboriginal participants^(^ ^44^ ^)^ and other populations^(^ ^45^ ^,^ ^46^ ^)^ with principles to guide implementation^(^ ^47^ ^–^ ^49^ ^)^.

We are aware of no other studies conducted in remote Indigenous Australia that have systematically examined mediators and moderators of dietary behaviour change. The authors of a multi-component study conducted in an Aboriginal community in remote Western Australia in the late 1990s posed that greater motivation and sense of control may have been associated with dietary improvement^(^ ^22^ ^)^; however, no assessment of these mediators was carried out.

Strengths of this study were that the study population was drawn from five communities spread across the NT that were randomly selected, thus giving confidence of the generalisability of the results to other like remote NT communities. Our well-balanced questionnaire was modified from previously validated questionnaires and piloted in the study population. Employment and training of community residents assisted with interpretation and helped ensure that the study was conducted in accordance with cultural practice to maximise engagement and boost participant’s confidence to respond. A further strength is the comprehensive evaluation of dietary intake. Efforts were made to increase data validity through reference to a 2-week calendar and significant events, use of visual aids to give guidance on usual intake and through standardised scripted information. The daily per capita mean intakes of fruit (75 g) and vegetable (87 g) were comparable with those reported by the 2012/2013 National Aboriginal and Torres Strait Islander Nutrition and Physical Activity survey for remote living Indigenous Australians (82 and 108 g, respectively)^(^ ^14^ ^)^. On the other hand, regular soft drink consumption (80 ml) was substantially less than total soft drink consumption reported for remote-living Indigenous Australians (390 ml), which probably reflects our cohort demographic of mostly middle-aged women, many of whom were non-consumers.

There are limitations to this study that need consideration in interpreting the findings. The attrition we experienced exceeded our expectation of 25 %. Unlike the Aboriginal Birth Cohort study^(^ ^31^ ^)^, we did not have the resources to follow-up participants who were not present in the community at the time of data collection and did not anticipate the level of mobility that we experienced. Our retention rate was similar to that reported by Flego *et al.* in relation to a cooking skills programme in Australia^(^ ^46^ ^)^. A high loss to follow-up is an issue for studies conducted among very mobile populations over lengthy periods. Attrition in our study is likely to have not affected the results; however, it further reduced study power. Besides the scientific results, this study provides a sense of how to assess mediators of behaviour change and dietary intake, and indicates expected retention, which can inform future larger studies. It will also help with power calculations for future studies. Our study was constrained by the lack of power to detect statistical differences in outcomes with time and in association with the mediators/moderators. A retrospective power calculation indicated that with 85 people who participated at T2, we had 80 % power to see a minimum difference of 30 g (equivalent to 1/3 sd) per person per day (as per protocol) in self-reported fruit and vegetable intake at T2 compared with the baseline intake (with paired *t* test and an *α* significance level of 0·05). Owing to the lack of a control group, we were not able to conduct formal tests of mediation. The benefit of a control group in such an exploratory study would need to outweigh the extensive resources (time and cost) required and burden to the community when participants also are asked to provide data and do not receive the immediate benefits of the intervention. It is extremely expensive to conduct research in the remote Indigenous community setting owing to geographic remoteness and the time needed to build relationships with communities. It is not unusual either for some communities to have several studies being conducted simultaneously, all seeking the participation of members. Last, we cannot dismiss the potential effect on dietary behaviour of the customer survey itself and that multiple statistical comparisons were made, thereby increasing the chance of showing a statistically significant result. The reduction in soft drink reported at T3 may have been an intervention effect or the result of increased attention being given to soft drink consumption through the administration of the survey or the result of retailers potentially increasing their effort to contribute to dietary improvement as a result of the SHOP@RIC study.

### Implications for research and practice

The high cost of food in the context of low incomes in the remote Indigenous communities remains a pertinent issue. The findings of this study suggest that the SHOP@RIC price discount/consumer education intervention on diet modified perceptions of fruit and vegetable affordability and had a better chance of affecting post intervention those who perceived fruit to be affordable than those who did not. It was not strong enough to overcome the constraints of food insecurity, barriers and low self-efficacy to cook and try new vegetables. This suggests that even in a context of socio-economic disadvantage, the more disadvantaged people are least likely to benefit from interventions unless their specific needs are considered.

In conclusion, our findings show that our combined intervention went someway to improving perceived affordability of vegetables and potentially fruit, but was likely not strong enough to overcome other mediators and moderators constraining behaviour change. This suggests that it will be challenging to achieve meaningful dietary improvement in remote Indigenous Australia at a population level without addressing the underlying constraints that reinforce unhealthy dietary behaviours. This requires long-term investment of government and political commitment. This study shows that steps towards this are likely to include addressing the high cost of food, alongside a mix of strategies that provide monetary incentive to increase purchase of healthier foods and address barriers to healthy eating, and enhance self-efficacy to cook and try new vegetables through programmes that are culturally appropriate. These need to be considered as part of a comprehensive approach to improving diet^(^ ^50^ ^)^.

## Supplementary material

For supplementary material accompanying this paper visit http://dx.doi.org/10.1017/S0007114518000880. click here to view supplementary material
