# Supplementary material for: Mediators and moderators of nutrition intervention effects in remote Indigenous Australia
Source: Br J Nutr. 2018 Jun 28;119(12):1424–33. doi: 10.1017/S0007114518000880 (PMC6088544; doi:10.1017/S0007114518000880)
Supplement: Supplementary file 1 [file S0007114518000880sup001.docx]

**Extra Material**

**Supplementary Table 1. Questions to assess mediators and moderators with response options and variable constructs**

| Mediator | Question | Response Options | Variable construct |
| --- | --- | --- | --- |
| Perceived fruit affordability | Price affect: When you do your shopping, does the price of food affect what you buy? | 1. I don’t look at the price, I just buy it; 2. I look at the price, but don’t worry and just buy it; 3. I sometimes use the price to buy it or leave it; 4. I often use the price to buy it or leave it; 5. I always use the price to buy it or leave it | Fruit perceived affordable if responded that price does not affect food/ drink purchases (i.e., price affect = 1 or 2) and the price of fruit is considered to be good or OK (i.e., think about price =1 or 2) |
|  | Think about price: What do you think about the price of fruit? | 1. Good; 2. OK; 3. Sometimes OK; 4. Too high; 5. Always too high |  |
| Perceived vegetable affordability | Price affect: When you do your shopping, does the price of food affect what you buy? | 1. I don’t look at the price, I just buy it; 2. I look at the price, but don’t worry and just buy it; 3. I sometimes use the price to buy it or leave it; 4. I often use the price to buy it or leave it; 5. I always use the price to buy it or leave it | Vegetables perceived affordable if responded that price does not affect food/ drink purchases (i.e., price affect = 1 or 2) and the price of vegetables is considered to be good or OK (i.e., think about price =1 or 2) |
|  | Think about price: What do you think about the price of vegetables? | 1. Good; 2. OK; 3. Sometimes OK; 4. Too high; 5. Always too high |  |
| Self-efficacy to positively change intake | Do you think you can eat more fruit each day? | Yes; No | Self-efficacy to eat more fruit if response=yes |
|  | Do you think you can eat more vegetables each day? | Yes; No | Self-efficacy to eat more vegetables if response=yes |
|  | Do you think you can drink more water each day? | Yes; No | Self-efficacy to drink more water if response=yes |
|  | Do you think you can drink less soft drink?^ | Yes definitely; Yes; Unsure; No; No definitely not | Self-efficacy to drink less soft drink if response=yes definitely (non-drinkers not included) |
| Self-efficacy to cook and try new vegetables | Cook: How do you feel about cooking with vegetables? | 1.I feel really sure; 2. I feel only a little bit sure; 3. I need help; 4. I don’t know how; 5. I don’t cook with vegetables | High self-efficacy to cook and try new vegetables if response to all three questions = 1 (I feel really sure) |
|  | Recipe: How do you feel about trying new ways of cooking with vegetables using a recipe? | 1. I feel really sure; 2. I feel only a little bit sure; 3. I need help; 4. I don’t know how; 5. I don’t cook with recipes |  |
|  | Try new: How would you feel about trying new vegetables you haven’t tasted before? | 1. I feel really sure; 2. I feel only a little bit sure; 3. I am not sure; 4. I would not try new vegetables; 5. I don’t eat vegetables |  |
| Perceived new knowledge (fruit/ vegetables)^#^ | From all of this (the SHOP@RIC strategy) did you learn anything new about fruit and vegetables? | Yes; No | New knowledge (fruit/vegetables) if response=yes |
| Perceived new knowledge (drinks) ^#^ | From all of this (the SHOP@RIC strategy) did you learn anything new about drinks? | Yes; No | New knowledge (drinks) if response=yes |
| Moderators | **Question** | **Response Options** | **Variable construct** |
| Food security | In the last 12 months, were there any times that you ran out of food, and couldn't afford to buy more? | Yes; No | Food secure if response to both questions = yes |
|  | Do you run out of money and just have foods like bread and tea to eat?^¥^ | Yes; No |  |
| Barriers | Which barriers stop you from eating more fruit and/or vegetables? | I eat enough^±^; I don’t have enough money to buy more; if I buy more everyone eats it; I don’t have a fridge; I don’t have a knife | These were recoded to a dichotomous barrier score of either no barriers (those who reported already eating enough) vs barriers |

^ Question not asked to participants who reported to not consume regular soft drink

^#^ Data collected at T2 only

^¥^ This question was devised based on our previous formative work that had identified bread and tea as staples for people in remote communities during times of constrained finances

^±^ ‘I already eat enough’ was coded as ‘no barriers’

**Supplementary Table 2. Percentage responses by survey question at T1, T2 and T3**

| **Summary Mediator/Moderator** | **Survey question** | **Survey responses** | **T1 (%)** | **T2 (%)** | **T3 (%)** |
| --- | --- | --- | --- | --- | --- |
| **Perceived fruit affordability** | **Price affect**: When you do your shopping, does the price of food affect what you buy? | I don’t look at the price, I just buy it | 22.3 | 22.4 | 11.0 |
|  |  | I look at the price, but don’t worry and just buy it | 31.1 | 25.9 | 27.4 |
|  |  | I sometimes use the price to buy it or leave it | 27.0 | 34.1 | 38.4 |
|  |  | I often use the price to buy it or leave it | 6.8 | 10.6 | 5.5 |
|  |  | I always use the price to buy it or leave it | 12.8 | 7.1 | 17.8 |
|  | **Think about price fruit**: What do you think about the price of fruit? | Good | 23.7 | 49.4 | 26.0 |
|  |  | OK | 26.4 | 18.8 | 19.2 |
|  |  | Sometimes OK | 25.7 | 24.7 | 26.0 |
|  |  | Too high | 14.9 | 4.7 | 17.8 |
|  |  | Always too high | 9.5 | 2.4 | 11.0 |
| **Perceived vegetable affordability** | **Think about price veg**: What do you think about the price of fruit? | Good | 23.7 | 49.4 | 34.3 |
|  |  | OK | 21.0 | 32.9 | 20.6 |
|  |  | Sometimes OK | 27.0 | 14.1 | 27.4 |
|  |  | Too high | 21.6 | 2.4 | 9.6 |
|  |  | Always too high | 6.8 | 1.2 | 8.2 |
| **Self-efficacy to positively change intake** | Do you think you can eat more fruit each day? | No | 8.8 | 11.8 | 37.0 |
|  |  | Yes | 91.2 | 88.2 | 63.0 |
|  | Do you think you can eat more veg each day? | No | 8.8 | 15.3 | 32.9 |
|  |  | Yes | 91.2 | 84.7 | 67.1 |
|  | Do you think you can eat more water each day? | No | 7.4 | 11.8 | 23.3 |
|  |  | Yes | 92.6 | 88.2 | 76.7 |
|  | Do you think you can drink less regular soft drink each day? | Yes definitely | 3.8 | 13.1 | 18.0 |
|  |  | Yes | 74.3 | 57.4 | 50.0 |
|  |  | Unsure | 4.8 | 11.5 | 14.0 |
|  |  | No | 15.2 | 16.4 | 16.0 |
|  |  | No definitely not | 1.0 | 1.6 | 2.0 |
|  |  | Missing | 1.0 | 0.0 | 0.0 |
| **Self-efficacy to cook and try new vegetables** | Cook: How do you feel about cooking with vegetables? | I feel really sure | 82.4 | 81.2 | 74.0 |
|  |  | I feel only a little bit sure | 10.8 | 15.3 | 20.6 |
|  |  | I need help | 6.1 | 3.5 | 5.5 |
|  |  | I don’t cook with vegetables | 0.7 | 0.0 | 0.0 |
|  | Recipe: How do you feel about trying new ways of cooking with vegetables using a recipe? | I feel really sure | 50.7 | 41.2 | 24.7 |
|  |  | I feel only a little bit sure | 18.2 | 17.7 | 11.0 |
|  |  | I need help | 10.1 | 5.9 | 8.2 |
|  |  | I don’t know how | 2.7 | 3.5 | 0.0 |
|  |  | I don’t cook with recipes | 18.2 | 31.8 | 56.2 |
|  | Try new: How would you feel about trying new vegetables you haven’t tasted before? | I feel really sure | 44.6 | 31.8 | 34.3 |
|  |  | I feel only a little bit sure | 24.3 | 38.8 | 38.4 |
|  |  | I am not sure | 13.5 | 16.5 | 11.0 |
|  |  | I would not try new vegetables | 17.6 | 12.9 | 16.4 |
|  |  | I don’t eat vegetables |  |  |  |
| **Perceived new knowledge (fruit/ vegetables)** | From all of this (the SHOP@RIC strategy) did you learn anything new about fruit and/or vegetables? | No | - | 56.5 | - |
|  |  | Yes | - | 43.5 | - |
| **Perceived new knowledge (drinks)** | From all of this (the SHOP@RIC strategy) did you learn anything new about drinks? | No | - | 64.7 | - |
|  |  | Yes | - | 35.3 | - |
| **Food security** | In the last 12 months, were there any times that you ran out of food, and couldn't afford to buy more? | No | 24.3 | 37.7 | 31.5 |
|  |  | Yes | 75.7 | 62.4 | 68.5 |
|  | If yes, how often did this happen? | Once per week | 39.9 | 27.1 | 37.0 |
|  |  | Once every two weeks | 23.7 | 24.7 | 16.4 |
|  |  | Once per month | 7.4 | 4.7 | 6.9 |
|  |  | Less than once per month | 4.7 | 5.9 | 8.2 |
|  |  | Don’t know | 24.3 | 37.7 | 31.5 |
|  | Do you run out of money and just have foods like bread and tea to eat? | No | 16.2 | 23.5 | 20.6 |
|  |  | Yes | 83.8 | 76.5 | 79.5 |
|  | If yes, how often did this happen? | Once per week | 59.7 | 50.8 | 58.6 |
|  |  | Once every two weeks | 26.6 | 35.4 | 24.1 |
|  |  | Once per month | 5.7 | 7.7 | 5.2 |
|  |  | Less than once per month | 4.0 | 1.5 | 8.6 |
|  |  | Don’t know | 4.0 | 4.6 | 3.5 |
| **Barriers** | What stops you from eating more fruit? | | | | |
|  | I eat enough fruit | No | 79.7 | 82.4 | 72.6 |
|  |  | Yes | 20.3 | 17.7 | 27.4 |
|  | I don’t have enough money to buy more | No | 32.4 | 35.3 | 34.3 |
|  |  | Yes | 67.6 | 64.7 | 65.8 |
|  | If I buy fruit everyone eats it | No | 60.1 | 72.9 | 76.7 |
|  |  | Yes | 39.9 | 27.1 | 23.3 |
|  | I don’t have a fridge | No | 87.8 | 88.2 | 89.0 |
|  |  | Yes | 12.2 | 11.8 | 11.0 |
|  | I don’t have a knife | No | 99.3 | 98.8 | 100 |
|  |  | Yes | 0.7 | 1.2 | 0.0 |
|  | Other |  | 0 | 0 | 0 |
|  | Which of these is the most important thing that stops you from having more fruit each day? | I eat enough | 15.4 | 11.8 | 23.3 |
|  |  | I don’t have enough money to buy more | 56.6 | 57.7 | 57.5 |
|  |  | If I buy fruit, everyone eats it | 17.5 | 12.9 | 8.2 |
|  |  | I don’t have a fridge | 7.0 | 4.7 | 8.2 |
|  |  | I don’t have a knife or way to cook it | 0.0 | 1.2 | 0.0 |
|  |  | Other | 3.5 | 11.8 | 2.7 |
|  | What stops you from eating more vegetables? | | | | |
|  | I eat enough | No | 77.7 | 74.1 | 71.2 |
|  |  | Yes | 22.3 | 25.9 | 28.8 |
|  | I don’t have enough money to buy more | No | 31.8 | 40.0 | 37.0 |
|  |  | Yes | 68.2 | 60.0 | 63.0 |
|  | If I buy more everyone eats it | No | 73.0 | 80.0 | 82.2 |
|  |  | Yes | 27.0 | 20.0 | 17.8 |
|  | I don’t have a fridge | No | 88.5 | 88.2 | 90.4 |
|  |  | Yes | 11.5 | 11.8 | 9.6 |
|  | I don’t have a knife or way to cook it | No | 95.3 | 96.5 | 100.0 |
|  |  | Yes | 4.7 | 3.5 | 0.0 |
|  | Other |  | 0 | 0 | 0 |
|  | Which of these is the most important thing that stops you from having more vegetables each day? | I eat enough | 21.9 | 21.2 | 24.7 |
|  |  | I don’t have enough money to buy more | 58.2 | 51.8 | 56.2 |
|  |  | If I buy more everyone eats it | 11.0 | 10.6 | 8.2 |
|  |  | I don’t have a fridge | 7.5 | 5.9 | 8.2 |
|  |  | I don’t have a knife or way to cook it | 0 | 0 | 0 |
|  |  | I don’t know how to cook vegetables | 0 | 0 | 0 |
|  |  | Other | 1.4 | 10.6 | 2.7 |

**Supplementary Table 3. Associations between the log-intake of each of fruit, vegetables and water and the dichotomous variables for mediators and moderators expressed in terms of percent difference relative to the reference group, regardless of time.**

|  | **Mean Gr (95% CI)** | **% difference (95% CI)** | **p** |
| --- | --- | --- | --- |
| **Fruit** |  |  |  |
| Fruit perceived not affordable | 74 (62, 89) | Ref |  |
| Fruit perceived affordable | 72 (58, 89) | -3.6 (-21.4,14.2) | 0.69 |
| Low self-efficacy to eat more fruit | 76 (51, 113) | Ref |  |
| High self-efficacy to eat more fruit | 70 (58, 84) | -7.8 (-43.9,28.3) | 0.67 |
| Barriers^≠^ | 72 (61, 86) | Ref |  |
| No barriers^φ^ | 79 (61, 102) | 9.2 (-15.6,33.9) | 0.47 |
| Food insecure | 72 (61, 86) | Ref |  |
| Food secure | 81 (62, 106) | 11.8 (-15.6,39.3) | 0.40 |
| **Vegetables** |  |  |  |
| Vegetable perceived not affordable | 89 (69, 110) | Ref |  |
| Vegetable perceived affordable | 89 (68, 118) | -2.6 (-20.0,25.3) | 0.82 |
| Low self-efficacy to eat more vegetables | 108 (73, 161) | Ref |  |
| High self-efficacy to eat more vegetables | 81 (69, 96) | -25.0 (-56.3,6.3) | 0.12 |
| Not high self-efficacy to cook and try vegetables | 79 (64, 98) | Ref |  |
| High self-efficacy to cook and try vegetables | 140 (104, 188) | 77.2 (31.5,123.0) | 0.001 |
| Barriers^≠^ | 80 (65, 97) | Ref |  |
| No barriers^φ^ | 122 (93, 160) | 53.2 (16.9,89.4) | 0.004 |
| Food insecure | 79 (65, 95) | Ref |  |
| Food secure | 149 (111, 201) | 89.4 (35.9,142.9) | 0.001 |
|  |  |  |  |
|  | **Mean mls (95% CI)** |  |  |
| **Water** |  |  |  |
| Low self-efficacy to drink more water | 832 (634, 1092) | Ref |  |
| High self-efficacy to drink more water | 942 (868, 1023) | 13.3 (-18.9, 45.4) | 0.42 |
| Food insecure | 963 (887, 1044) | Ref |  |
| Food secure | 981 (832, 1157) | 1.9 (-15.9,19.7) | 0.84 |
|  |  |  |  |
|  | **Median mls (IQR)** |  |  |
| **Soft drinks-including non-drinkers** |  |  |  |
| Low self-efficacy to drink less soft drink | 53.6 (1, 201) | Ref |  |
| High self-efficacy to drink less soft drink | 50.0 (1, 267) | -16.3 (-34.7,7.2) | 0.16 |
| Food insecure | 40.0 (1, 201) | Ref |  |
| Food secure | 49.0 (1, 179) | 11.7 (-7.8,31.2) | 0.54 |

^≠^ Barriers to increasing fruit intake include : i) having enough money to buy more; ii) having to provide for many people in a household; not having a refrigerator and not having a knife for preparation. The same barriers apply to vegetable intake

^φ^ The response category ‘I already eat enough’ was coded as ‘no barriers’ for the questions about barriers for both fruit and vegetables
